# Altered Functional Connectivity in Patients With Sloping Sensorineural Hearing Loss

**DOI:** 10.3389/fnhum.2019.00284

**Published:** 2019-08-22

**Authors:** Tomasz Wolak, Katarzyna Cieśla, Agnieszka Pluta, Elżbieta Włodarczyk, Bharat Biswal, Henryk Skarżyński

**Affiliations:** ^1^Institute of Physiology and Pathology of Hearing, Bioimaging Research Center, World Hearing Center, Warsaw, Poland; ^2^Faculty of Psychology, University of Warsaw, Warsaw, Poland; ^3^Department of Biomedical Engineering and Department of Radiology, New Jersey Medical School, NJIT, Newark, NJ, United States

**Keywords:** functional connectivity, resting state, sensorineural hearing loss, partial deafness, neuronal plasticity

## Abstract

**Background:**

Sensory deprivation, such as hearing loss, has been demonstrated to change the intrinsic functional connectivity (FC) of the brain, as measured with resting-state functional magnetic resonance imaging (rs-fMRI). Patients with sloping sensorineural hearing loss (SNHL) are a unique population among the hearing impaired, as they have all been exposed to some auditory input throughout their lifespan and all use spoken language.

**Materials and Methods:**

Twenty patients with SNHL and 21 control subjects participated in a rs-fMRI study. Whole-brain seed-driven FC maps were obtained, with audiological scores of patients, including hearing loss severity and speech performance, used as covariates.

**Results:**

Most profound differences in FC were found between patients with prelingual (before language development, PRE) vs. postlingual onset (after language development, POST) of SNHL. An early onset was related to enhancement in long-range network connections, including the default-mode network, the dorsal-attention network and the fronto-parietal network, as well as in local sensory networks, the visual and the sensorimotor. A number of multisensory brain regions in frontal and parietal cortices, as well as the cerebellum, were also more internally connected. We interpret these effects as top-down mechanisms serving optimization of multisensory experience in SNHL with a prelingual onset. At the same time, POST patients showed enhanced FC between the salience network and multisensory parietal areas, as well as with the hippocampus, when they were compared to those with PRE hearing loss. Signal in several cortex regions subserving visual processing was also more intra-correlated in POST vs. PRE patients. This outcome might point to more attention resources directed to multisensory as well as memory experience. Finally, audiological scores correlated with FC in several sensory and high-order brain regions in all patients.

**Conclusion:**

The results show that a sloping hearing loss is related to altered resting-state brain organization. Effects were shown in attention and cognitive control networks, as well as visual and sensorimotor regions. Specifically, we found that even in a partial hearing deficit (affecting only some of the hearing frequency ranges), the age at the onset affects the brain function differently, pointing to the role of sensitive periods in brain development.

## Introduction

The brain develops the capability of a complex form based on its genetic makeup, that is modified by its sensory experience (Kral and Eggermont, 2007; Ballantyne et al., 2008). The structural and functional organization of the brain occurs throughout life as a result of repeated engagement within specific neural systems to process ongoing information (Guerra-Carrillo et al., 2014). For some functions, however, such as developing certain auditory, visual, and language abilities, there seems to exist sensitive developmental periods during which the nervous system is especially responsive to certain incoming stimuli (Daw, 2009; Friederici, 2012; Gordon et al., 2013; Kral, 2013). If stimulation is disrupted in these early life periods, for instance due to sensory deprivation, the neuronal organization might become irreversibly altered (Cardon et al., 2012; Kral and Sharma, 2012; Voss, 2013; Friedmann and Rusou, 2015; Gordon et al., 2015).

One MRI technique, which may serve as a window into experience-related brain development and plasticity, particularly, is resting state functional magnetic resonance imaging (rs-fMRI). It measures low-frequency spontaneous fluctuations of blood oxygen level-dependent (BOLD) signals across the whole brain. This effect was first described by Biswal et al. (1995), Zhang and Raichle (2010). Rs-fMRI benefits from the fact that the brain at rest retains its inherent functional organization (also termed, functional connectivity, FC) and thus temporal correlations between various brain regions are hypothesized to reflect prior coincidental neural firing.

Several canonical resting state networks (RSN) can be reproducibly identified using a variety of analysis approaches (Smitha et al., 2017). These include networks that are enhanced during the performance of specific tasks, such as the salience network (SN) for selection of useful information from the environment, the fronto-parietal (FPN) network involved in task monitoring, the dorsal (DAN) and ventral (VAN) attention networks, the language network, as well as several sensory networks (e.g., visual, auditory, sensorimotor). The only RS network that decreases its activity during attention-demanding tasks is the default mode network (DMN), underlying unconstrained, conscious cognition (Greicius, 2008).

Sensory deprivation resulting from blindness or deafness provides an excellent opportunity to study experience-dependent brain plasticity (Finney et al., 2001; Bock and Fine, 2014; Sharma et al., 2015). Similar to other conditions leading to adverse behavioral performance (Kelly et al., 2008), including aging and neurodegenerative diseases (Hohenfeld et al., 2018) as well as mental disorders (Azeez and Biswal, 2017), sensory deprivation has been widely demonstrated to change intrinsic FC (in auditory deficits: Burton et al., 2012; Maudoux et al., 2012; Schmidt et al., 2013; Husain et al., 2014; Li et al., 2015; Liu et al., 2015; Ding et al., 2016; Sabbah et al., 2016; Hofmeier et al., 2018; Luan et al., 2019a, b; Rosemann and Thiel, 2019; Xu et al., 2019c, b; in visual deficits: Burton et al., 2014).

Nevertheless, only scarce literature exists on brain resting brain network changes in patients with moderate to severe hearing loss, as is the case in the current study (Schmidt et al., 2013; Husain et al., 2014; Puschmann and Thiel, 2017; Xu et al., 2019c). The findings include increased functional couplings between frontal and parietal regions (Schmidt et al., 2013, Husain et al., 2014) and within the parietal cortex (Schmidt et al., 2013), decreased (Xu et al., 2019a) or increased (Schmidt et al., 2013) FC from insula to the inferior parietal cortex, decreased (Husain et al., 2014) or increased (Puschmann and Thiel, 2017) signal correlations between auditory and visual cortices, decreased FC between the insula and sensorimotor as well as high-order frontal brain areas (Xu et al., 2019c), and increased or decreased couplings between frontal and visual or sensorimotor sensory regions (Puschmann and Thiel, 2017 and Husain et al., 2014, respectively).

Although some convergence can be seen in the published findings, suggesting the involvement of additional cognitive resources as well as possible enhanced collaboration between sensory regions indicating compensatory plasticity in hearing loss, the outcomes of the existing studies are hardly comparable. Besides the fact that diverse patient populations have been studied (in terms of the accompanying tinnitus, age, etiology of HL) and they were mostly very limited in size, the above-mentioned experiments selected only specific networks for the analysis [e.g., only auditory, DMN and DAN in Schmidt et al. (2013) and in Husain et al. (2014); visual cortex in Puschmann et al. (2014)]. In addition, a variety of distinct methods and statistical thresholds were applied to quantify connectivity.

The purpose of the current study was to examine the effect of an auditory deficit in all RSNs. This seemed to be a particularly valid endeavor since deafness has become perceived as a “connectome disease,” i.e., affecting whole brain function, such as with cognitive and emotional processing, far beyond the auditory system (Cieśla et al., 2016; Kral et al., 2016). Specifically, we investigated a population with mid- to high-frequency sensorineural hearing loss (SNHL; also termed *partial deafness/PD*, Skarzynski et al., 2010). In addition, we explored whether measures of functional network organization between *various* brain areas are correlated with audiological performance in the patient group, including the severity of HL and speech understanding scores.

The population selected for this study was unique in that they represented patients with both prelingual hearing loss (congenital or developed early, before language acquisition) and postlingual hearing deficits which have, however, never been complete. All patients had been exposed to some acoustic input throughout their lifetime and all used spoken language. For this very specific patient population, we had the following hypotheses: (1) we expected to see most FC alterations in other brain regions but not the auditory network *per se*, which has never been completely deprived of its natural input (as opposed to the congenitally profoundly deaf patients, Li et al., 2016) we expected FC to be differently affected in the two patient subpopulations. Besides the existing rs-fMRI literature, these hypotheses were derived from our clinical experience, as well as our findings in a similar patient population (Wolak et al., 2017) who showed unchanged tonotopic organization (frequency representation) in the auditory cortex except for a slight enlargement of the low-frequency regions in the early-deprived group. Furthermore, as also mentioned above, there is a vast body of research showing that early sensory deficits affect brain function differently than the acquired ones which is related to the existence of sensitive periods in development (although the most tested populations were either completely deaf or completely blind) (Kral and ODonoghue, 2010; Wan et al., 2010; Desgent and Ptito, 2012; Kupers and Ptito, 2014; Slimani et al., 2014; Friedmann and Rusou, 2015; Wong et al., 2015; Kral et al., 2017).

## Materials and Methods

### Subjects

The study was approved by the Medical Ethics Committee of the Institute of Physiology and Pathology of Hearing (IPPH) in Kajetany/Warsaw, Poland. Participants gave their written informed consent prior to the study and received no monetary reward for their participation. Twenty patients (11F, 9M; age 34.9 ± 8.2 years, range: 16.25–47.50 years) were recruited from the Institute of Physiology and Pathology of Hearing. Ten patients had higher (university degree), eight medium (high-school) and two basic (primary school) education. All patients had idiopathic sloping sensorineural hearing loss (partial deafness, Skarzynski et al., 2010). Ten patients had prelingual HL (diagnosed before the age of 3 years; PRE) and 10 acquired it above the age of 7 years (postlingually; POST). The mean age of the PRE group was (32.3 ± 9.4 years; age range: 16.25–42.30) and of the POST group was (37.6 ± 6.2 years; age range: 26.92–47.50) [Mann Whitney *U* test; *z* = −1.32; *p* = 0.19] and involved fewer women (*N* = 3) than the POST group (*N* = 8) [Chi^2^ = 5; *p* = 0.025]. All subjects were right-handed. The control group included 12 women and 9 men, aged 31.8 ± 7.2 years and was matched for education levels (8 medium, 13 higher). All subjects in the control group had normal hearing (<25 dB for 0.125–8 kHz) and no tinnitus. Please see further details of participants in Table 1.

**TABLE 1:** Demographic and audiological data of the patient group.

**No**	**Sex**	**Age**	**Onset of**	**Duration of**	**Tinnitus**	**Hearing aids**
		**(years)**	**SNHL**	**SNHL (years)**	**0–no**	**0–none**
					**1–yes**	**1–one**
						**2–two**
1	M	31	PRE	–	0	1
2	F	37.5	PRE	–	0	2
3	F	30.3	PRE	–	0	2
4	F	41.9	PRE	–	0	0
5	M	26.92	PRE	–	0	0
6	F	31.08	PRE	–	0	0
7	M	17.25	PRE	–	0	1
8	M	42.3	PRE	–	1	0
9	M	36.8	PRE	–	1	2
10	M	36.6	PRE	–	1	2
11	F	45.25	POST	18	0	0
12	F	42.17	POST	22	0	0
13	M	16.25	POST	3	0	2
14	F	37.25	POST	8	0	0
15	F	32.9	POST	28	1	0
16	M	32.17	POST	14	1	0
17	F	42.17	POST	6	1	0
18	M	47.5	POST	19	1	1
19	F	37.5	POST	9	1	1
20	M	34.7	POST	19	1	2

### Audiometric Evaluation

**FIGURE 1:**
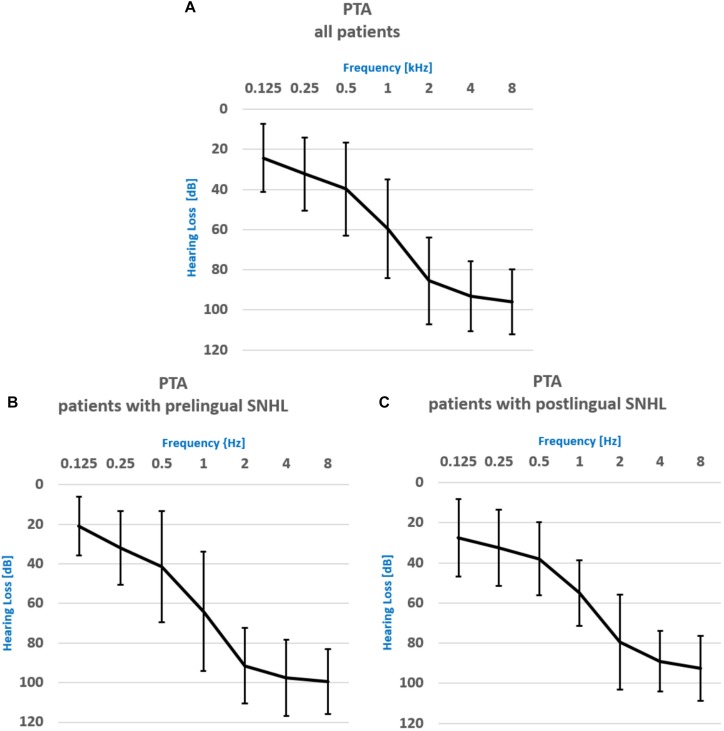
Pure Tone Audiometry results for **(A)** the whole patient group, **(B)** the patient group with prelingual onset of SNHL, **(C)** the patient group with postlingual onset of SNHL.

A comprehensive audiometric evaluation was performed for each subject, both patients and the normal hearing individuals (NH) controls. The average pure tone audiometry for the whole group of patients for frequency ranges from 500 Hz to 2000 Hz (PTA) was 56.3 ± 24.3 dB for the left ear and 59.8 ± 24.4 dB for the right ear (see the audiogram for all frequency ranges in Figure 1). Word Recognition Scores (WRSs) were also obtained, with the Polish Monosyllabic Pruszewicz test (Pruszewicz et al., 1999) (speech presented at 65 dB) and were on the average 49 ± 38% and 44 ± 34% for the right and the left ear, respectively. The higher the score, the more words the patient was able to understand. All patients used spoken language supported with lip-reading to communicate. The PTA and the WRS scores for the PRE and the POST group were 66 ± 21 dB and 11 ± 14%, 58 ± 18 dB and 75 ± 17%, respectively. WRSs were statistically different between groups (Man Whitney *U* test, *z* = −3.77; *p* = 0.000).

### Psychological Evaluation

All subjects took part in a professional psychological interview to make sure that they would be able to participate in the study. As the applied psychological tools were described elsewhere in detail (see section “Materials and Methods” in Cieśla et al., 2016), they are only briefly mentioned here. Polish versions of two screening tools, the beck depression inventory (BDI) and the state-trait-anxiety-inventory (STAI) were used to assess depressive and anxiety symptoms in study participants. BDI is a self-report inventory with 21 multiple-choice items to be responded to on a 0–3 point scale (the maximum total is 63 points) (Zawadzki et al., 2009). The STAI form X has 40 questions, with 20 questions measuring anxiety as a *state* and 20 referring to levels of anxiety as a personal *trait*. The participant responds him/herself on a 4-point Likert-type scale and the maximum total score is 80 (Wrześniewski, 2011). With both questionnaires, higher scores indicate more severe symptoms.

### Rs-fMRI Data Acquisition

RS-fMRI data were acquired at the Biomedical Imaging Center of IPPH in Kajetany/Warsaw, Poland. RS-fMRI examination was conducted using a 3T Siemens TRIO TIM scanner equipped with a 12-channel head matrix coil. The parameters of the EPI sequence were the following: time of repetition (TR) = 2000 [ms]; time of echo (TE) = 30 [ms]; flip angle (FA) = 90°; voxel size = 3 × 3 × 3 mm; imaging matrix = 64 × 64; no of slices = 41; time of acquisition (TA) = 8.08 min; 250 data points. Participants were instructed to relax during scanning with their eyes closed and not to think of anything in particular. A structural T1 MR sequence had the following parameters: TR = 1900 [ms]; TE = 2.26 [ms]; time of inversion (TI) = 900 [ms]; flip angle (FA) = 9°; field of view (FOV) = 28.8 × 27.0 [cm]; matrix = 320 × 300; voxel size = 0.9 × 0.9 × 0.9 [mm]; pixel bandwidth = 200 Hz/pix; no of slices = 208; TA = 5:11 min.

### Data Pre-processing

A standard preprocessing pipeline was applied in CONN (Connectivity Toolbox) which uses functions from Statistical Parametric Mapping 12 (SPM12) software^1^. Preprocessing of the functional data included slice timing correction, motion correction, scrubbing, linear detrending, band-pass filtering (0.01 Hz < f < 0.1 Hz), co-registration to individual T1 structural scans, spatial normalization to MNI space, and spatial smoothing (6 mm Gaussian kernel). Each subject’s structural scan was segmented into gray matter, white matter, and cerebrospinal fluid (CSF) tissue classes using the unified segmentation approach implemented in SPM12. In addition, the Artifact Detection Tool^2^ was used to measure motion artifacts in all individuals. Linear regression of confounding effects was applied including: mean signal from white matter and CSF, motion parameters obtained in the realignment step, volumes that showed movement exceeding 0.5mm from the scrubbing step (in the ART toolbox with conservative settings, 95th percentile in a normative sample) and 10 first scans (effect of rest). It was found that the mean frame-wise displacement (±SD) was: 0.112 ± 0.038 mm in the NH and 0.128 ± 0.056 mm in patients with SNHL (groups were not different in that aspect, *p* = 0.283). Seven individual volumes required scrubbing (both groups together).

### Statistical Analysis

All subsequent analysis of rs-fMRI outcomes was performed in CONN-fMRI FC toolbox ver. 17f^3^. The first-level whole-brain seed-driven FC maps were obtained by estimating temporal correlations of BOLD signal in seeds corresponding to nodes of canonical RSN. In addition, we used nodes corresponding to the Heschl gyri obtained from the new default atlas (132 ROIs) combining the FSL Harvard-Oxford atlas cortical and subcortical areas and the AAL atlas cerebellar areas. The analysis was performed separately for each node (the list and coordinates of all seeds is presented in Supplementary Table S1) and for both the patient and the normal hearing group.

For the patient group, to measure bivariate correlations between the connectivity strength and patients’ audiological scores, including WRS and PTA, seed-to-voxel correlation coefficients (Pearson’s R correlation coefficients) were computed and converted to normally distributed *z*-scores using Fisher’s transform. We used WRS and PTA as covariates in the same model as they were not correlated with one another (Kendall’s tau, *p* = 0.49). Since the hearing loss severity and speech understanding scores were comparable for both ears in all participants, averaged binaural PTA and WRS were used for the correlation analysis.

A second-level random effects analysis was then applied to create within-group statistical parametric maps (SPMs) for each RS network and to examine connectivity differences between groups, i.e., between the normal hearing subjects and patients, as well as between patient groups with prelingual onset of SNHL and postlingual onset of SNHL. In addition, we performed a comparison analysis between patients with tinnitus and without tinnitus, as well as with and without hearing aids. This was also done due to the fact that the proportion of patients with and without tinnitus/hearing aids was different in the PRE and the POST subgroup. For all these comparisons mean binaural PTA and WRS values were used as covariates. SPMs were generated for each outcome and thresholded at the voxel level at *p* < 0.001 uncorrected and then the cluster-size FWE-corrected level of *p* < 0.05 and with the Holm–Bonferroni method at *p* < 0.05 to account for the effect of comparisons between multiple networks and regions (Holm, 1979).

The outcomes of psychological questionnaires (STAI, BDI) were analyzed with SPSS ver. 20 software.

## Results

### Psychological Questionnaires

The normal hearing and the patient group were not different in terms of their scores in BDI (Patients: 8.5 ± 7.2 points; NH group: 5 ± 5 points) and STAI (Patients: *anxiety as state* 32 ± 7.7 and *anxiety as trait* 32.4 ± 7.4; NH group: *anxiety as trait* 28.1 ± 5.5 and *anxiety as trait* 29.5 ± 6.2) (*p* > 0.05). In both groups the symptoms indicated non-existent or only mild depressive and anxiety symptoms.

### Rs-fMRI Data

#### Brain-Behavior Relationship

Tables 2, 3 present RS functional connections that were found correlated with audiological scores (PTA, WRS) in the whole patient group (thresholded at the cluster-size FWE-corrected level of *p* < 0.05 and Holm–Bonferroni level at *p* < 0.05 to account for multiple comparisons of FCs). All functional connections that were found correlated with PTA and WRS values at FWEc < 0.05 but failed to exceed the Holm–Bonferroni *p* < 0.05 threshold are presented in Supplementary Tables S2a,b.

**TABLE 2:** Functional connections found correlated with binaural PTA scores in the whole patient group.

		**Main anatomical**	**Size of the target**	**MNI coordinates**			***p*-value FWE_c_*p* < 0.05**	
	**RS network and**	**region in the**	**region in voxels**	**of the target**			**corr. and Holm–Bonferroni**	**Direction of**
	**seed region**	**target region**	**(2 × 2 × 2 mm)**	**region (X,Y,Z)**			***p* < 0.05 corr.**	**correlation**
1.	Visual medial	Occipital pole L	170	−14,	−102,	−10	0.000103	positive
2.	Visual medial	Occipital pole R	128	16,	−106,	0	0.001014	positive
3.	Dorsal attention FEF L	Precuneus	144	−6,	−68,	32	0.000369	positive
4.	Sensorimotor sup.	Occipital Fusiform Gyrus L	113	−36,	−80,	−14	0.004052	negative
5.	Language pSTG R	Postcentral Gyrus Left	107	−12,	−40,	54	0.003978	negative
6.	DMN MPFC	Precentral Gyrus R	106	60,	−6,	28	0.005222	negative

*FEF, frontal eye fields; Sup, superior; pSTG, posterior superior temporal gyrus; DMN, default mode network; MPFC, medial prefrontal cortex; L, left;, R, right.*

**TABLE 3:** Functional connections found correlated with binaural WRS scores in the whole patient group.

		**Main anatomical**	**Size of the target**	**MNI coordinates**			***p*-value FWE_c_*p* < 0.05**	
	**RS network and**	**region in the**	**region in voxels**	**of the target**			**corr. and Holm–Bonferroni**	**Direction of**
	**seed region**	**target region**	**(2 × 2 × 2 mm)**	**region (X,Y,Z)**			***p* < 0.05 corr.**	**correlation**
1.	Heschl Gyrus L	Lingual Gyrus/Intracalcarine Cortex L/R	134	4,	−78,	0	0.000676	positive

*L, left; R, right.*

Hearing severity (binaural PTA) was found positively correlated with FC between the visual medial seed and bilateral occipital poles, as well as the left frontal eye field and the precuneus. At the same time, negative correlations (the less hearing loss severity the more functional coupling) between the superior node of the sensorimotor network and the occipital fusiform gyrus, as well as the right superior temporal gyrus of the language network and the DMN prefrontal hub to postcentral gyrus left and precentral gyrus right, respectively.

The only statistically significant correlation that was found for binaural WRS values in patients was a positive one between the left Heschl gyrus and bilateral lingual/intracalcarine cortex.

### Subjects With SNHL vs. Normal Hearing Individuals

**FIGURE 2:**
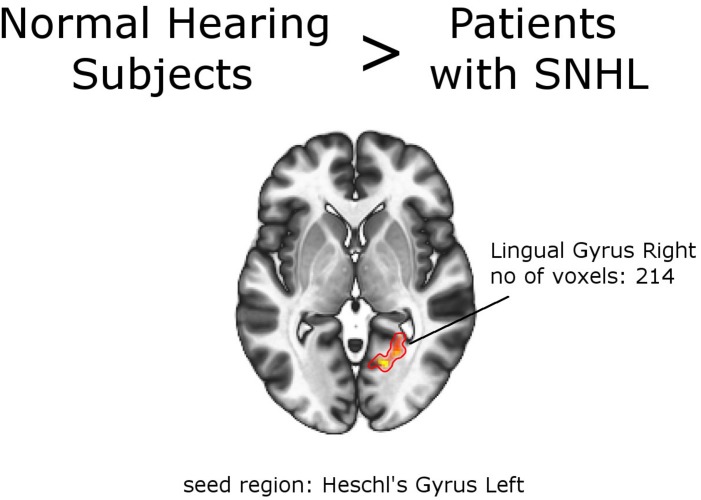
Between-group effects. Increased functional connectivity in normal hearing subjects, compared to the group of patients as a whole. Networks and seed regions are indicated below each slice. The target regions of altered FC are outlined in red for better visualization. The number of voxels is the size of the whole target region, and the name indicates the anatomical area the majority of the voxels belonged to. If the brain side of the target region is not stated, it was in both hemispheres.

**FIGURE 3:**
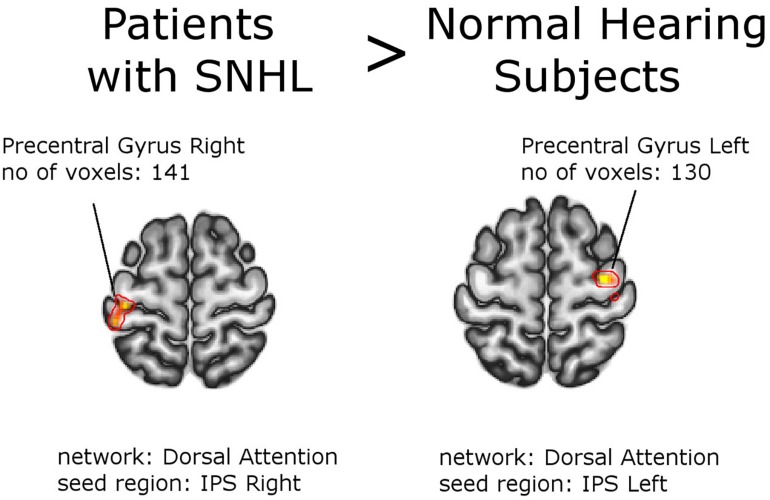
Between-group effects. Increased functional connectivity in the group of patients as a whole, compared to normal hearing subjects. Networks and seed regions are indicated below each slice. The target regions of altered FC are outlined in red for better visualization. The number of voxels is the size of the whole target region, and the name indicates the anatomical area the majority of the voxels belonged to. If the brain side of the target region is not stated, it was found in both hemispheres.

Second-level analysis revealed stronger FC between left Heschl gyrus and right lingual gyrus in NH, compared to the whole group of patients with SNHL (thresholded at the cluster-size FWE-corrected level of *p* < 0.05 and Holm–Bonferroni corrected at *p* < 0.05). The reverse comparison showed decreased FC in the normal hearing group between bilateral inferior parietal sulci, and bilateral precentral gyri. With this stringent statistical approach there were no other functional connections that showed significant between-group differences (Figures 2, 3 and Tables 4, 5). All networks and seeds tested in this comparison are listed in Supplementary Table S1. All functional connections that were found different between the groups at FWE_c_ cluster size corrected threshold of *p* < 0.05 but failed to exceed the Holm–Bonferroni *p* < 0.05 correction are presented in Supplementary Tables S3a,b.

**TABLE 4:** Increased functional connectivity in Normal Hearing Subjects vs. Patients with SNHL (with PTA and WRS values used as regressors).

		**Main anatomical**	**Size of the target**	**MNI coordinates of the**			***p*-value FWE_c_*p* < 0.05**
	**RS network and**	**region in the**	**region in voxels**	**target region**			**corr. and Holm–Bonferroni**
	**seed region**	**target region**	**(2 × 2 × 2 mm)**	**(X,Y,Z)**			***p* < 0.05 corr.**
1.	Heschl Gyrus L	Lingual Gyrus R	214	16,	−64,	0	0.000020

*L, left; R, right.*

**TABLE 5:** Increased functional connectivity in Patients with SNHL vs. Normal Hearing Subjects (with PTA and WRS valuesused as regressors).

		**Main anatomical**					
		**region in the**	**Size of the target**	**MNI coordinates of the**			***p*-value FWE_*rm c*_*p* < 0.05**
	**RS network and**	**target region**	**region in voxels**	**target region**			**corr. and Holm–Bonferroni**
	**seed region**	**(L, left; R, right)**	**(2 × 2 × 2 mm)**	**(X,Y,Z)**			***p* < 0.05 corr.**
1.	Dorsal attention IPS R	Precentral Gyrus R	141	32,	−14,	62	0.001142
2.	Dorsal attention IPS L	Precentral Gyrus L	130	−38,	−26,	66	0.001859

*IPS, intra parietal sulcus; L, left; R, right.*

### Patients With Prelingual vs. Postlingual Onset of Snhl

**FIGURE 4:**
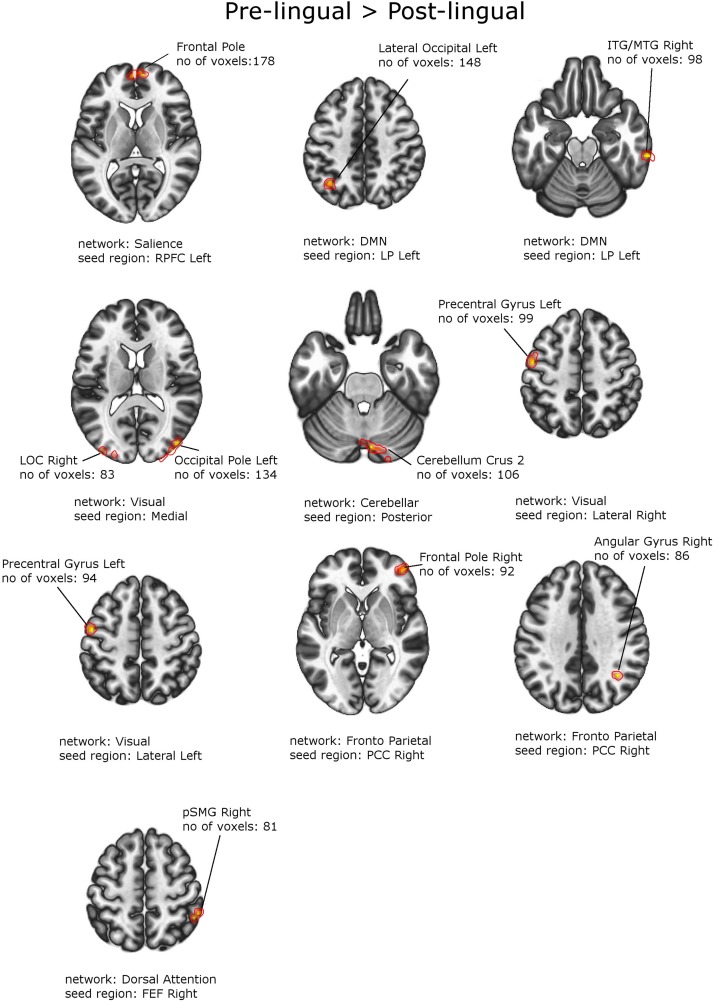
Between-group effects. Increased functional connectivity in patients with Prelingual SNHL vs. Postlingual SNHL. Networks and seed regions are indicated below each slice. The target regions of altered FC are outlined in red for better visualization. The number of voxels is the size of the whole target region, and the name indicates the anatomical area that the majority of the voxels belonged to. If the brain side of the target region is not stated, it was found in both hemispheres.

**FIGURE 5:**
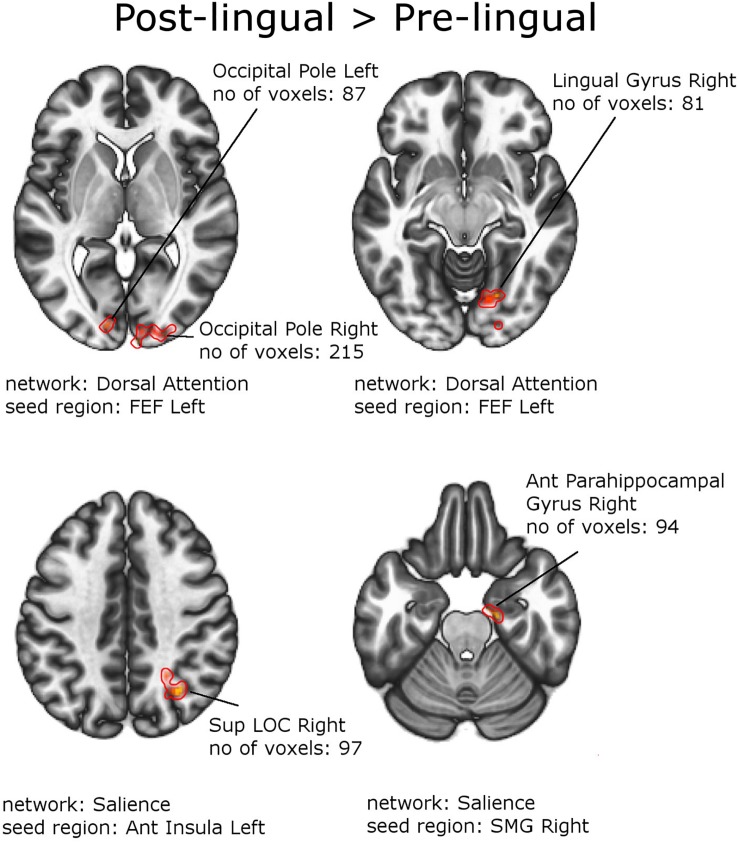
Between-group effects. Increased functional connectivity in patients with Postlingual SNHL vs. Prelingual SNHL. Networks and seed regions are indicated below each slice. The target regions of altered FC are outlined in red for better visualization. The number of voxels is the size of the whole target region, and the name indicates the anatomical area the majority of the voxels belonged to. If the brain side of the target region is not stated, it was in found both hemispheres.

Figures 4, 5 and Tables 6, 7 depict differences in FC between subpopulations of patients with early *vs.* late onset of SNHL. In short, the analysis showed that subjects with prelingual SNHL exhibit a pattern of increased connectivity within the following canonical RS networks: salience, FPN, DMN, cerebellar, DAN, and visual. In the visual network, specifically, subjects with prelingual SNHL onset demonstrated increased connectivity between the medial visual seed and the occipital pole bilaterally, as well as between lateral visual cortex and left precentral gyrus. At the same time patients with postlingual onset of their hearing loss showed increased connectivity in DAN and SN, as well as some visual areas.

**TABLE 6:** Increased functional connectivity in patients with prelingual onset of SNHL, compared to patients with postlingual onset of SNHL (with PTA and WRS values used as regressors).

		**Main anatomical**	**Size of the target**	**MNI coordinates of the**			***p*-value FWE_c_*p* < 0.05**
	**RS network and**	**region in the**	**region in voxels**	**target region**			**corr. and Holm–Bonferroni**
	**seed region**	**target region**	**(2 × 2 × 2 mm)**	**(X,Y,Z)**			***p* < 0.05 corr.**
1.	Salience RPFC L	Frontal pole L/R	178	8,	60,	8	0.000029
2.	DMN	Lateral Occipital L	148	−38,	−60,	46	0.000151
	LP L	ITG/MTG R	98	64,	−30,	−22	0.003416
3.	Visual	Occipital Pole L	134	−22,	−98,	28	0.000251
	Medial	LOC R	83	44,	−82,	14	0.007649
4.	Celebellar posterior	Cerebellum Crus 2	106	6,	−78,	−28	0.001823
5.	Visual lateral R	Precentral Gyrus L	99	−50,	−8,	52	0.002987
6.	Visual lateral L	Precentral Gyrus L	94	−50,	−6,	52	0.004205
7.	Fronto parietal PCC R	Frontal Pole R	92	44,	50,	0	0.004536
		Angular Gyrus R	86	36,	−54,	36	0.006909
8.	Dorsal attention FEF R	pSMG R	81	50,	−46,	58	0.009263

*RPFC, rostral prefrontal cortex; PCC, posterior cingulate cortex; DMN, default mode network; ITG, inferior temporal gyrus; MTG, middle temporal gyrus; LP, lateral parietal cortex; FEF, frontal eye fields; IFG, inferior frontal gyrus, LOC, lateral occipital complex; pSMG, posterior supramarginal gyrus; OFusG, occipital fusiform gyrus; L, left; R, right.*

**TABLE 7:** Increased functional connectivity in the patient group with postlingual onset of SNHL, compared to patients with prelingual onset of SNHL (with PTA and WRS values used as regressors).

		**Main anatomical**	**Size of the target**	**MNI coordinates of the**			***p*-value FWE_c_*p* < 0.05**
	**RS network and**	**region in the**	**region in voxels**	**target region**			**corr. and Holm–Bonferroni**
	**seed region**	**target region**	**(2 × 2 × 2 mm)**	**(X,Y,Z)**			***p* < 0.05 corr.**
1.	Dorsal attention FEF L	Occipital Pole R	215	22,	−88,	−6	0.000002
		Occipital Pole L	87	−12,	−90,	−2	0.005764
3	Dorsal attention FEF L	Lingual Gyrus R	81	20,	−70,	−10	0.008903
4	Salience ant insula L	Sup LOC R	97	34,	−60,	40	0.003958
5	Salience SMG R	Ant Parahippocampal Gyrus R	94	18,	−16,	−24	0.004775

*Sup LOC, superior lateral occipital complex; Ant, anterior; Sup, superior; DMN, default mode network; FEF, frontal eye fields; IFG, inferior frontal gyrus; SMG, supramarginal gyrus; L, left; R, right.*

Supplementary Table S1 depicts all tested networks and seed regions that were used for comparisons between outcomes in patients with early vs. late onset of SNHL. Supplementary Tables S4a,b show all FCs that were found different between patient subpopulations at threshold of FWE_c_ *p* < 0.05 but failed to exceed the Holm–Bonferroni *p* < 0.05 correction.

Outcomes of the comparison analysis between patients with and without tinnitus, as well as with and without hearing aids were presented in Supplementary Tables S5a–S6b. None of the detected connectivity differences overlapped with those detected between patients with prelingual vs. postlingual onset of hearing loss.

## Discussion

In the current study we investigated brain resting-state connectivity in patients with sloping sensorineural hearing loss. We found that a number of functional connections were modulated by the audiological scores presented by the patients, including their hearing loss severity and speech recognition scores. Furthermore, in a comparison analysis, significant effects were demonstrated between patients with an early onset of SNHL (prelingual) and those who acquired their HL later in life (postlingual). We found differences in both directions, but mostly increased functional couplings in the patient group with early auditory deficits. These results are very interesting as they may reflect the neuronal effects of the onset of a partial hearing impairment *per se*. Meanwhile, both patient subpopulations did experience some degraded auditory input, but just until different moments in their lifetime. As patients with prelingual onset of SNHL and those with postlingual onset of SNHL included different proportion of those who experienced tinnitus and/or wore hearing aids, we also checked how the two latter factors affect FC. We found no connectivity differences that could be attributed to both the onset of SNHL and tinnitus/using hearing aids. In all analyses comparing subpopulations of patients we accounted for the variance in FC related to the severity of hearing loss and speech understanding scores, in order not to confound the results. Furthermore, patient subgroups were matched for sex, as well as age for the representation of patients with tinnitus. Interestingly, against our expectations, the analysis of RSNs revealed a significant effect involving the auditory network, and namely an increase in FC in the normal hearing subjects, as compared to the patient group as a whole, between early auditory and early visual cortices.

### Increased Functional Connectivity in Patients With Prelingual SNHL

We found in patients with prelingual SNHL increased functional couplings both in long-ranging brain networks, such as the DMN, the FPN and the DAN, as well as in local sensory networks, i.e., the visual areas and the sensorimotor areas. We saw that most of the FC alterations involved ipsilateral connections (*vs.* cross-hemisphere), with the effect present more often in the right brain hemisphere. We suggest in this patient population a mechanism of top-down modulation by the FPN axis. This mechanism serves to obtain optimal integration of inputs, including aspects of speech, coming from multiple sensory modalities.

In the current study we found in patients with an early (partial) auditory deprivation enhanced intrinsic FC within the visual network *–* including a number of early and association visual subregions. This effect was accompanied by more functional couplings from multisensory brain regions, such as the SMG (underlying visuo-tactile integration) and AG (underlying audio-visual integration) in inferior parietal cortex, and the inferior temporal gyrus (ITG). The ITG is, among other functions, involved in recognizing visual patterns, also those with linguistic features (Ardila et al., 2015). These findings point to possible multisensory compensation mechanisms occurring in the early auditory deprived brain. Indeed, there are many studies demonstrating that especially patients with early sensory deficits, both visual and auditory, rely more on multisensory experiences than the healthy population. It has even been shown that deprived sensory regions, such as the auditory cortex in congenital profound deafness, can engage in the analysis of inputs coming from other modalities (see, e.g., Karns et al., 2012 for touch and Vachon et al., 2013 for vision) or high-order functions, such as processing language (Bavelier et al., 2006; Campbell et al., 2007; Xia et al., 2017). In the current experiment we do not claim that auditory cortex became involved in processing of inputs from other modalities because we did not find enhanced connectivity between the auditory network and other sensory or association brain regions.

We suggest that the demonstrated enhanced collaboration between the multisensory and “unisensory” brain areas in this specific hearing impaired population reflects, (a) processing of vibrotactile and spatial aspects of acoustic signal, such as people’s gestures or music-induced vibration, (b) experience with lip-reading and face feature analysis, (c) enhanced sensory-motor-visual integration (Verger et al., 2017; Xie et al., 2019). The latter is suggested in some animal works, which report increased input to sensorimotor areas and more sensorimotor-auditory integration after cochlear damage (Hofmeier et al., 2018; Xu et al., 2019b). We can, therefore, speculate that an early onset of HL requires more reliance on motor and visual aspects of speech but also maybe monitoring of one’s own speech which is more challenging due to the limited auditory feedback (Bavelier et al., 2006; Xia et al., 2017; Xu et al., 2019b). From our clinical experience we can confirm that early auditory deprivation affects speech production, in terms of features, such as voicing and articulation, as well as the rhythm, even if only partially (Szkielkowska et al., 2000).

In the PRE patients we also saw bilateral frontal poles having increased FC with dorsolateral prefrontal cortex. The latter is one of the main hubs of the SN, which serves environment monitoring and information integration. Frontal pole, due to its dense structural connectivity with many cortical and subcortical brain regions, has been proposed to participate in the highest level of unification of information coming from all sensory systems, as well as in processes such as attention and memory, decision making, novelty detection and multiple-task coordination (Powell and Voeller, 2004; Hartogsveld et al., 2018). We also found, in patients whose hearing abilities degraded early in their lifetime, increased FC between frontal brain regions (frontal poles, frontal eye fields) and parietal brain regions, including PCC, supramarginal (SMG) and angular gyri (AG). Both SMG and AG have been demonstrated in a number of tasks engaging control processing and shifting of attention (see Dye and Hauser, 2014 for a review).

We suggest the following underpinnings of all the described effects; In patients with a prelingual onset of SNHL, the incoming auditory input, including speech, is degraded from the very early stages of life and thus requires increased effort and attention to understand (Rosemann and Thiel, 2019). At the same time, the available cognitive resources nare limited and, thus, they need to converge on trying to decipher the incoming acoustic inputs. Meanwhile, the environment constantly provides multiple competing stimuli that can act as distractors (Knudsen, 2007). Indeed, it has been shown that patients with hearing loss use more frontal and parietal cortices when performing auditory tasks (Husain et al., 2014), with the FPN axis suggested to be involved in cognitive aspects of auditory processing (Chen et al., 2017). As in normal hearing subjects, cognitive control is probably executed by the same largely non-overlapping brain networks in the hearing-impaired individuals, including the SN as well as the FPN network (Luan et al., 2019b).

In addition, the cerebellum was more intrinsically interconnected in the PRE patient population. The cerebellum is now recognized to play a major role in sensorimotor and cognitive processes, such as phonological fluency, semantic word association and even metalinguistic skills (De Smet et al., 2013; Xu et al., 2019b). There exist both feedforward and feedback connections between the cerebellum and the cerebrum, including the auditory structures, which suggests the possible modulatory effect of the cerebellum both at the level of subcortical as well as on the cortical stages of information processing (Chen et al., 2016; Feng et al., 2018).

In sum we suggest that the control mechanisms described above, involving the FPN axis and maybe the cerebellum as well, serve to optimize integration of multisensory (visual and somatosensory) aspects of the incoming stimulation, with the degraded auditory input or regardless of it (we saw no changes in the auditory network).

### Increased Functional Connectivity in Patients With Postlingual SNHL

Patients with acquired hearing deficits, in turn, revealed more functional couplings between various visual subregions and the frontal node of the DAN (with early visual regions) – the frontal eye field, as well as in the SN (with higher visual regions). In this subpopulation the effects were relatively balanced in terms of the left and right hemisphere as well as ipsi- vs. contra-lateral changes in FC. These outcomes suggest that some specific functional changes in neuronal processing might accompany postlingual partial hearing deficits, which are slightly distinct to those that might characterize patients with early hearing loss onset.

The role of the frontal eye field (FEF), which is on the border with the motor-related precentral gyrus, is control of visual attention and eye movement. Interestingly, it has been shown that FEF is densely and reciprocally structurally connected with the visual cortex (Schall, 2009). Whether, the increased FC in the current study between FEF and multiple visual subregions reflects the existing structural connections requires further examination, with techniques such as Diffusion Tensor Imaging (DTI; Soares et al., 2013).

Besides the enhanced FEF-visual connections we also saw increased functional couplings between lateral parts of the visual network and insula, in patients with postlingual onset of SNHL. The lateral occipital cortex is a mid-level visual cortex area subserving object recognition. Interestingly, a recent study showed increased engagement of LOC in a visual verbal rhyming task in cochlear implant candidates with postlingual profound deafness (Lazard and Giraud, 2017). In addition, although traditionally viewed as a high-level visual area, LOC was found to develop strengthened FC to the auditory cortex after auditory–tactile sensory substitution training (learning to recognize shapes from sounds that describe them) (Kim and Zatorre, 2011). This outcome suggested LOC as a brain area that is actually available to all sensory systems and not only the visual one.

The tested postlingual subpopulation of patients also demonstrated increased FC between another Silence Network region, the multisensory and attention-related SMG, with the hippocampus. As cortico-hippocampal interaction was demonstrated to be implicated in successful memory formation (Ranganath et al., 2005) this finding may indicate reliance on representations of earlier completely normal hearing experience in patients who acquired a partial hearing deficit later in life.

In sum, we can speculate on the following compensatory mechanism occurring in late onset SNHL. The Silence Network (including the insula and the SMG) that was found to be more strongly internally coupled in patients with postlingual SNHL, integrates multiple internal and external stimuli (Xu et al., 2019b). An auditory deficit can affect functioning of SN as it deprives it of one crucial source of sensory inputs. This in turn affects decision making and directing attention. Indeed, anterior insula was shown to engage in top-down detection of silent events and attention, including the auditory and the visual modality (Amaral and Langers, 2015; Xu et al., 2019b). Here, we suggest that in a situation of SNHL that was acquired later in life, SN directs attention to the remaining memory traits of speech as well as the existing current visual (and maybe also tactile) aspects of speech signal. Due to the increased involvement of the FEF and a number of visual subregions, we speculate that in partial postlingual SNHL it is mainly the audio-visual multisensory integration that helps the patients engage in the environment, despite their disability.

### Brain-Behavior Correlations

In the whole patient population, after we applied a very stringent statistical threshold, we found that increased severity of hearing loss correlated with increased FC strength between the left medial visual cortex and the left occipital pole. Both these regions can be considered the early visual cortex. We therefore hypothesize that subjects with more severe deafness (higher PTA) have to rely on visual information more when recognizing speech signal, as for example during lip-reading, recognizing facial expressions or gestures. Coordinates of regions that showed increased FC were almost the same as those found more connected in the patient subgroup with prelingual SNHL (vs. postlingual SNHL). This might suggest that although the PTA values in both patient populations were not statistically different, the fact that the PTA was slightly lower in the early-onset group (66 ± 21 dB in PRE vs. 58 ± 18 in POST) might have had some effect on RSN.

Another connection that was positively correlated with PTA was between FEF and precuneus, which might again imply an increased reliance on/attention to the incoming visual input in patients with partial hearing loss. At the same time, with better hearing (lower PTA values) FC was enhanced between sensorimotor brain areas (superior parietal cortex, pre- and postcentral gyri) and multisensory brain regions, including STG, PFC and higher visual cortex. This again suggests that collaboration of senses might serve as compensation for the degradation of the incoming auditory input. In addition, we saw that with increasing speech recognition scores FC between the early auditory cortex (HG) and the early visual cortex (lingual gyrus) was also enhanced. This finding of enhanced connections between early sensory cortices is indeed intriguing, as we also found that this coupling was stronger in the normal hearing population as compared to the patient group as a whole (see below). For more in-depth interpretation of these finding, due to the relatively small sample size of patients (*N* = 20), further investigation is essential.

### Comparisons of Functional Connectivity Between Patients With SNHL and Normal Hearing Individuals

In the patient population we saw enhanced FC between bilateral IPS in the DAN and bilateral sensorimotor cortices. This internal correlation of regions underlying perceptuo-motor processing and coordination again point to the fundamental importance of multisensory processing in the hearing deprived CNS, during processes such as reading and producing gestures, as well as one’s own speech production control. More interestingly, however, participants with normal hearing demonstrated enhanced collaboration between early auditory and visual seeds (see above for the same effect of increased FC accompanying increased speech understanding in the patient population). We did not hypothesize that we would see any effects in the auditory system of the tested patients as they never experienced a total auditory deprivation. experienced joint exposure. However, we speculate that in case of normal hearing what we see is the effect of the very often of early sensory cortices to auditory and visual input, both not degraded. This experience might enhance functional coupling between early sensory brain regions. In the case of patients, especially those with early developed auditory deficits, such an experience is never the case. This does not exclude, however, enhanced collaboration between higher sensory and association regions in the hearing impaired. In fact, with only partial hearing deficit the early auditory cortex is never fully deprived of auditory input.

### Methodological Issues and Study Limitations

#### A Whole-Brain Approach

In the current study it was especially important for us to take a whole-brain approach to the analysis of RSN. At the same time, in all but few recent studies involving patients with hearing deficits (Puschmann and Thiel, 2017; Chen et al., 2018; Xie et al., 2019), the RSN analysis was limited to only several selected resting-state networks (as a whole or parts of them), mainly auditory, DMN, VAN/DAN, and SN. We understand the rationale of testing the involvement of certain pre-selected brain regions in deprivation-related functional brain changes. In the current study, however, although we put forward a hypothesis it had to be first verified, as we tested a unique patient population. In addition, in the face of the most recent studies in human and in animals, we assumed a perspective of hearing impairment as a connectome disease and, as such, something potentially related to changes in many remote brain networks (Xu et al., 2019c). The whole brain approach, however, makes the statistical thresholds more stringent (more comparisons imply more corrections for multiple comparisons). It is, therefore, possible that we might have found more differences, both between the patient population as a whole and the normal hearing subjects, as well as across patients, if we focused our analysis on a limited number of seeds and/or used a less stringent statistical approach. For completion, in the Supplementary Materials, we provide a list of all outcomes of the RS analysis that did not exceed the strict statistical threshold after correction for multiple comparisons between RSN/regions.

#### The Role of Tinnitus

We acknowledge that an estimated 30–40% of patients with SNHL also have tinnitus which can affect FC, as it was demonstrated in a number of studies (Burton et al., 2012; Schmidt et al., 2013; Chen et al., 2014, 2015; Minami et al., 2015, 2017; Leaver et al., 2016; Wei-Wei et al., 2018). Some studies also found changes in the cerebellum or between the cerebellum and the cortex in tinnitus (Chen et al., 2017; Feng et al., 2018). In our study half of the patients (*N* = 9) also suffered from tinnitus. We therefore cannot exclude the possibility that the presented outcomes are related to the presence of tinnitus. Indeed, we did find FC differences between patients with and without the experience of tinnitus. None of these differences overlapped with those found for comparisons between patients with early and late onset of SNHL. Studying the effect of tinnitus was not the aim of the current experiment and so we did not collect any further measures of tinnitus in individual subjects, such as tinnitus laterality, duration, severity and bothersomeness. Meanwhile, increasingly more rs-fMRI studies indicate that these parameters significantly change the way tinnitus affects brain FC (Schmidt et al., 2013; Zhang et al., 2015; Chen et al., 2017; Feng et al., 2018). Such an endeavor should, however, be definitely undertaken in our future studies.

#### The Studied Sample

Finally, the relatively small sample size in the current experiment should be mentioned as a study limitation. It is definitely the case that individual variability of FC can be high and thus can significantly influence group results if the studied groups is small. In addition, half of the study participants wore one or two hearing aids, with the other half not using any kind of amplification (which can affect FC, as shown in Supplementary Materials). In addition, there were different proportions of patients with and without tinnitus in the PRE and POST patient subpopulation. We are planning, in our future studies, to evaluate RSNs in homogenous subgroups of patients separately, for instance involving only patients with an early or a late onset of the hearing impairment, and/or patients that have similar levels of hearing loss and/or speech performance, users and non-users of HAs, with and without tinnitus. These parameters can then be more reliably evaluated as potential factors shaping resting state FC.

## Conclusions

Taken together, the results of our study indicate that both a general loss of function and compensatory plasticity likely coexist between various networks in the auditory-deprived brain. Whether or not sensory deprivation caused by SNHL might be solely attributed to disruptions in connectivity patterns is, nevertheless, yet to be determined. However, earlier partial hearing deficits definitely seem to induce increase in FC particularly, such as by the additional involvement of subregions of the visual and sensorimotor systems. In sum we propose a mechanism of top-down modulation of perceptual processing by the frontal, parietal and cerebral brain regions that supports optimal integration of incoming multisensory information. Nevertheless, further rs-MRI studies are needed to address the effects of partial hearing loss of varying severity and age at onset, on intrinsic functional networks in the brain.

## Data Availability

The datasets generated for this study are available on request to the corresponding author.

## Ethics Statement

This study was approved by the Bioethical Committee of the Institute of Physiology and Pathology of Hearing.

## Author Contributions

TW designed the fMRI paradigm, analyzed the fMRI data, and prepared the manuscript. KC designed the study, performed the fMRI study and psychological assessments, and prepared the manuscript. AP analyzed the fMRI data and prepared the manuscript. EW recruited the patients and performed the medical examinations. BB and HS consulted the study design and preparation of the manuscript.

## Conflict of Interest Statement

The authors declare that the research was conducted in the absence of any commercial or financial relationships that could be construed as a potential conflict of interest.

## Footnotes

1 http://www.fil.ion.ucl.ac.uk/spm/

2 https://www.nitrc.org/projects/artifact_detect

3 http://www.nitrc.org/projects/conn
