# Supplementary material for: Altered Functional Connectivity in Patients With Sloping Sensorineural Hearing Loss
Source: Front Hum Neurosci. 2019 Aug 22;13:284. doi: 10.3389/fnhum.2019.00284 (PMC6713935; doi:10.3389/fnhum.2019.00284)
Supplement: Supplementary file 1 [file Data_Sheet_1.docx]

**Supplementary Materials**

**Table S1.** List of RS networks and seeds with their coordinates. Seeds are defined from CONN's ICA analyses of the HCP dataset (497 subjects).

|  | **RS network and seed region** | **MNI Coordinates**  **X,Y,Z** |
| --- | --- | --- |
| 1. | DefaultMode.MPFC | 1, 55, -3 |
| 2. | DefaultMode.LP L | -39,-77, 33 |
| 3. | DefaultMode.LP R | 47,-67, 29 |
| 4. | DefaultMode.PCC | 1,-61, 38 |
| 5. | SensoriMotor.Lateral L | -55,-12, 29 |
| 6. | SensoriMotor.Lateral R | 56,-10, 29 |
| 7. | SensoriMotor.Superior | 0,-31, 67 |
| 8. | Visual.Medial | 2,-79, 12 |
| 9. | Visual.Occipital | 0,-93, -4 |
| 10. | Visual.Lateral L | -37,-79, 10 |
| 11. | Visual.Lateral R | 38,-72, 13 |
| 12. | Salience.ACC | 0, 22, 35 |
| 13. | Salience.AntInsula L | -44, 13, 1 |
| 14. | Salience.AntInsula R | 47, 14, 0 |
| 15. | Salience.RPFC L | -32, 45, 27 |
| 16. | Salience.RPFC R | 32, 46, 27 |
| 17. | Salience.SMG L | -60,-39, 31 |
| 18. | Salience.SMG R | 62,-35, 32 |
| 19. | DorsalAttention.FEF L | -27, -9, 64 |
| 20. | DorsalAttention.FEF R | 30, -6, 64 |
| 21. | DorsalAttention.IPS L | -39,-43, 52 |
| 22. | DorsalAttention.IPS R | 39,-42, 54 |
| 23. | FrontoParietal.LPFC L | -43, 33, 28 |
| 24. | FrontoParietal.PPC L | -46,-58, 49 |
| 25. | FrontoParietal.LPFC R | 41, 38, 30 |
| 26. | FrontoParietal.PPC R | 52,-52, 45 |
| 27. | Language.IFG L | -51, 26, 2 |
| 28. | Language.IFG R | 54, 28, 1 |
| 29. | Language.pSTG L | -57,-47, 15 |
| 30. | Language.pSTG R | 59,-42, 13 |
| 31. | Cerebellar.Anterior | 0,-63,-30 |
| 32. | Cerebellar.Posterior | 0,-79,-32 |
|  | | |
| 33. | Heschl Gyrus Left and Right* | |

MPFC (Medial Prefrontal Cortex), LP (Lateral Posterior), PCC (Posterior Cingulate Gyrus), ACC (Anterior Cingulate Gyrus), RPFC (Rostral Prefrontal Cortex), ANtInsula (Anterior Insula), SMG (Supramarginal Gyrus), FEF (Frontal Eye Field), IPS (IntraParietal Sulcus), LPFC (Lateral Prefrontal Cortex), PPC (Posterior Parietal Cortex), IFG (Inferior Frontal Gyrus), pSTG (posterior Superior Temporal Gyrus); *form the new default atlas (132 ROIs) combining the FSL Oxford-Harvard atlas cortical and subcortical areas; L – left, R-right

**Table S2a** Functional connections found correlated with binaural PTA scores in the whole patient group without the Holm-Bonferroni correction for multiple FC comparisons.

|  | **RS network and seed region** | **Main anatomical region in the target region** | **Size**  **of the target region in voxels**  **(2x2x2mm)** | **MNI coordinates of the target region (X,Y,Z)** | **p-value FWE_c_ cluster size corr. P<0.05** | **Direction of correlation** |
| --- | --- | --- | --- | --- | --- | --- |
| 1. | Dorsal Attention FEF L | sLOC L | 79 | -36, -80, -14 | 0.019123 | positive |
| 2. | Dorsal Attention IPS L | MTG/ITG | 89 | -34, -70, 36 | 0.012760 | positive |
| 3. | Cerebellar Posterior | Occipital Pole L | 78 | 60, -6, 28 | 0.027202 | positive |
| 4. | DMN MPFC | Precentral Gyrus L | 72 | -44, -12, 32 | 0.042129 | negative |

MPFC (Medial Prefrontal Cortex), FEF (Frontal Eye Field), IPS (IntraParietal Sulcus), DMN (Default Mode Network), MTG (Middle Temporal Gyrus), ITG (Inferior Temporal Gyrus), sLOC (superior Lateral Occipital Cortex); L – left, R-right

**Table S2b** Functional connections found correlated with binaural WRS scores in the whole patient group without the Holm-Bonferroni correction for multiple FC comparisons.

|  | **RS network and seed region** | **Main anatomical region in the target region** | **Size**  **of the target region in voxels**  **(2x2x2mm)** | **MNI coordinates of the target region (X,Y,Z)** | **p-value FWE_c_ cluster size corr. P<0.05** | **Direction of correlation** |
| --- | --- | --- | --- | --- | --- | --- |
| 1. | Visual Lateral L | Frontal Pole R | 75 | 20, 66, 16 | 0.030557 | positive |
| 2. | Salience ACC | supLOC R | 77 | 40, -60, 58 | 0.032056 | positive |
| 3. | Fronto Parietal LPFC L | Angular Gyrus R/ supLOC R | 72 | 50, -60, 24 | 0.038252 | positive |
| 4. | Salience SMG L | Postcentral Gyrus L | 71 | -8, -44, 72 | 0.039850 | positive |
| 5. | Cerebellar Posterior | Superior Frontal Gyrus L/R | 71 | 0, 24, 52 | 0.042450 | negative |

LPFC (lateral Prefrontal Cortex), ACC (Anterior Cingulate Gyrus), SMG (Supramarginal Gyrus), supLOC (superior Lateral Occipital Cortex); L – left, R-right

**Table S3a** Increased functional connectivity in Normal Hearing Subjects vs Patients with SNHL (with PTA and WRS values used as regressors) without the Holm-Bonferroni correction for multiple FC comparisons.

|  | **RS network and seed region** | **Main anatomical region in the target region** | **Size**  **of the target region in voxels**  **(2x2x2mm)** | **MNI coordinates of the target region (X,Y,Z)** | **p-value FWE_c_ cluster size corr. p<0.05** |
| --- | --- | --- | --- | --- | --- |
| 1. | DMN.PCC | sLOC L | 122 | -36,-62,6 | 0.003028 |
| 2. | Salience.ACC | pCG | 107 | 4,-22,24 | 0.006234 |
| 3. |  | Precuneus | 92 | 20,-62,32 | 0.014765 |
| 4. | Salience.RPFC R | Occipital Pole R | 85 | 14,-88,30 | 0.024187 |

DMN (Default Mode Network), PCC (Posterior Cingulate Cortex), ACC (Anterior Cingulate Cortex), rPFC (Rostral Prefrontal COrtex), sLOC (superior Lateral Occipital Cortex), pCG(posterior Cingulate Gyrus); L – left, R-right

**Table S3b** Increased functional connectivity in Patients with SNHL vs Normal Hearing Subjects (with PTA and WRS values used as regressors) without the Holm-Bonferroni correction for multiple FC comparisons.

|  | **RS network and seed region** | **Main anatomical region in the target region** | **Size**  **of the target region in voxels**  **(2x2x2mm)** | **MNI coordinates of the target region (X,Y,Z)** | **p-value FWE_c_ cluster size corr. P<0.05** |
| --- | --- | --- | --- | --- | --- |
| 1. | Dorsal Attention IPS L | Precentral Gyrus R | 76 | 30,-22,68 | 0.039846 |
|  |  | Cerebellum6 R | 75 | 22,-58,-20 | 0.042382 |
| 2. | Visual Occipital | infLOC L | 84 | -46,-76,-6 | 0.024246 |
| 3. | Cerebellar Posterior | Insular Cortex R | 81 | 30,24,-6 | 0.031103 |
| 4. | sLOC L | Cerebellum 4 5 L | 80 | -8,-50,-14 | 0.034237 |

IPS (IntraParietal Sulcus), sLOC (superior Lateral Occipital Sulcus), Cerebellum 4,5,6 (4,5,6 crus), infLOC (inferior Lateral Occipital Cortex); L – left, R-right

**Table S4a** Increased functional connectivity in the patient group with prelingual onset of SNHL, compared to postlingual onset of SNHL (with PTA and WRS values used as regressors) without the Holm-Bonferroni correction for multiple FC comparisons.

|  | **RS network and seed region** | **Main anatomical region in the target region** | **Size of the target region in voxels**  **(2x2x2mm)** | **MNI coordinates of the target region (X,Y,Z)** | **p-value FWE_c_ cluster size corr. P<0.05** |
| --- | --- | --- | --- | --- | --- |
| 1. | Language IFG R | OFusG R | 70 | 20, -76, -12 | 0.021149 |
| 2. | Language IFG R | Lingual Gyrus L | 62 | -16, -72, -12 | 0.039204 |
| 3. | Visual Occipital | Occipital Pole R | 65 | 26, -98, 8 | 0.036720 |

IFG (Inferior Frontal Gyrus, OFusG(Occipital Fusiform Gyrus); L – left, R-right

**Table S4b** Increased functional couplings in the patient group with postlingual onset of SNHL, compared to prelingual onset of SNHL (with PTA and WRS values used as regressors) without the Holm-Bonferroni correction for multiple FC comparisons.

|  | **RS network and seed region** | **Main anatomical region in the target region** | **Size of the target region in voxel**  **(2x2x2mm)** | **MNI coordinates of the target region (X,Y,Z)** | **p-value FWE_c_ cluster size corr. P<0.05** |
| --- | --- | --- | --- | --- | --- |
| 1. | DMN LP L | Lateral Occipital R | 81 | 28, -84, 20 | 0.010999 |
| 2. | Cerebellar Posterior | Middle Temporal Gyrus R | 70 | 56, -54, -4 | 0.022839 |

DMN LP (Default Mode Network, lateral parietal); L – left, R-right

**Table S5a** Increased functional connectivity in the patient group with tinnitus, compared with the patient group without tinnitus (with PTA and WRS values used as regressors) with the Holm-Bonferroni correction for multiple FC comparisons.

|  | **RS network and seed region** | **Main anatomical region in the target region** | **Size of the target region in voxel (2x2x2mm)** | **MNI coordinates of the target region (X,Y,Z)** | **p-value FWEc cluster size corr. p<0.05** |
| --- | --- | --- | --- | --- | --- |
| 1 | Lateral Occipital Cortex R | Cerebellum Crus2 L | 129 | -12, -96, -32 | 0.000587 |
| 2 | Salience RPFC L | Middle Temporal Gyrus R | 93 | 70, -42, 2 | 0.004578 |
| 3 | Dorsal Attention FEF R | Pre/PostCentral Gyrus L/R | 89 | -8, -36, 76 | 0.005393 |

RPFC – rostral prefrontal cortex, FEF – frontal eye field; L – left, R - right

**Table S5b** Increased functional connectivity in the patient group without tinnitus, compared with the patient group with tinnitus (with PTA and WRS values used as regressors) with the Holm-Bonferroni correction for multiple FC comparisons.

|  | **RS network and seed region** | **Main anatomical region in the target region** | **Size of the target region in voxel (2x2x2mm)** | **MNI coordinates of the target region (X,Y,Z)** | **p-value FWEc cluster size corr. p<0.05** |
| --- | --- | --- | --- | --- | --- |
| 1 | Visual Lateral L | Lateral Occipital Cortex, superior division L/ Cuneal Cortex L | 112 | -6, -80, 26 | 0.001225 |
| 2 | Dorsal Attention IPS L | Lateral Occipital Cortex, superior division L | 110 | -24, -80, 52 | 0.001248 |
| 3 | Lateral Occipital Cortex R | Lateral Occipital Cortex ,superior division L | 105 | -38, -86, 24 | 0.002593 |
| 4 | Language IFG R | Cerebellum Crus2 R | 98 | 42, -60, -44 | 0.002833 |
| 5 | Language pSTG R | Middle/Superior Frontal Gyrus L | 88 | -34, 14, 54 | 0.006494 |
| 6 | Sensorimotor, Superior | Angular Gyrus R | 87 | 56, -52, 38 | 0.008924 |

IPS – Inferior Parietal Sulcus, IFG – Inferior Frontal Gyrus, pSTG – posterior Superior Temporal Gyrus; L – left, R – Right

**Table S6a** Increased functional connectivity in the patient group with Hearing Aids, compared with the patient group without Hearing Aids (with PTA and WRS values used as regressors) with the Holm-Bonferroni correction for multiple FC comparisons.

|  | **RS network and seed region** | **Main anatomical region in the target region** | **Size of the target region in voxel (2x2x2mm)** | **MNI coordinates of the target region (X,Y,Z)** | **p-value FWEc cluster size corr. p<0.05** |
| --- | --- | --- | --- | --- | --- |
| 1 | Default Mode LP R | Paracingulate Gyrus L / Frontal Medial Cortex | 164 | -6, 36, -10 | 0.000061 |
| 2 | Default Mode LP R | Paracingulate Gyrus R/L | 78 | 0, 28, 30 | 0.013718 |
| 3 | Salience ACC | Angular Gyrus R | 109 | 52, -56, 36 | 0.001791 |
| 4 | Default Mode LP L | Caudate L | 91 | -16, 18, 6 | 0.005615 |
| 5 | Default Mode MPFC | Lateral Occipital Cortex R | 77 | 38, -68, 32 | 0.013993 |

LP – lateral parietal, ACC – Anterior Cingulate Cortex, MPFC – medial Prefrontal Cortex; L – left, R – right

**Table S6b** Increased functional connectivity in the patient group without Hearing Aids, compared with the patient group with Hearing Aids (with PTA and WRS values used as regressors) with the Holm-Bonferroni correction for multiple FC comparisons.

|  | **RS network and seed region** | **Main anatomical region in the target region** | **Size of the target region in voxel (2x2x2mm)** | **MNI coordinates of the target region (X,Y,Z)** | **p-value FWEc cluster size corr. p<0.05** |
| --- | --- | --- | --- | --- | --- |
| 1 | Heschl’s Gyrus L | Precentral Gyrus R | 173 | 38, -70, -10 | 0.000026 |
| 2 | Salience ACC | Occipital Pole L | 126 | -24, -104, -6 | 0.000618 |
| 3 | Salience ACC | Supramarginal Gyrus, posterior division R | 81 | 56, -36, 14 | 0.011729 |
| 4 | Salience SMG R | Middle Frontal Gyrus R | 108 | 56, 22, 38 | 0.001873 |
| 5 | Default Mode MPFC | Cerebelum Crus2 L | 94 | -28, -86, -32 | 0.004225 |
| 6 | Salience AntInsula R | Cerebelum 6 L | 81 | -20, -56, -20 | 0.012413 |

ACC – anterior Cingulate Cortex, SMG – Supramarginal gyrus, MPFC – medial Prefrontal Cortex, AntInsula – anterior Insula

**Contribution to the field**

According to the WHO, almost 0.5 billion people worldwide have some level of disabling hearing loss (>40dB in the better ear). And the numbers are increasing, especially in the aging society. The most common types of hearing loss are deficits affecting middle and high frequency hearing (sloping hearing loss). This work complements the scarce existing knowledge on brain function in a patient population with a sloping hearing loss, by evaluating the brain at rest. The resting state has been shown to reflect prior experience of the individual, as the brain forms specific neuronal networks which spontaneously fluctuate at the same low frequency. We show altered functional connectivity between such networks in patients with sloping hearing loss, as compared to normal hearing subjects, which we find to be related to the age at onset of the impairment as well as its severity. Among others, we show changed organization of the attention and cognitive control networks, as well as the visual and sensorimotor areas. Due to the specific sensory experience of this population, i.e. both exposure to some acoustic input throughout their lifetime as well as using spoken language to communicate, these patients show unique patterns of compensation mechanisms that might in part improve their functioning. The most profound difference in functional connectivity between patients who developed hearing deficits early vs late underlies the role of sensitive periods in brain function development.
